# Inherent illnesses and attacks: an ethnographic study of interpretations of childhood Acute Respiratory Infections (ARIs) in Manhiça, southern Mozambique

**DOI:** 10.1186/1471-2458-11-556

**Published:** 2011-07-13

**Authors:** Lianne Straus, Khátia Munguambe, Quique Bassat, Sonia Machevo, Christopher Pell, Anna Roca, Robert Pool

**Affiliations:** 1 Barcelona Centre for International Health Research (CRESIB), Hospital Clinic/Institut d'Investigacions Biomediques, University of Barcelona, Rosselló 132, 08036 Barcelona, Spain; 2 Centro de Investigação em Saúde da Manhiça (CISM), Manhiça, CP 1929, Rua 12, Maputo, Moçambique; 3 Faculdade de Medicina, Universidade Eduardo Mondlane, Mozambique; 4 CIBER Epidemiología y Salud Pública (CIBERESP), Spain; 5 Centre for Global Health and Inequality, University of Amsterdam, OZ Achterburgwal 185, 1012 DK, Amsterdam, The Netherlands

## Abstract

**Background:**

Pneumonia is a leading cause of childhood hospitalisation and child mortality in Africa. This study explores local interpretations of Acute Respiratory Infections (ARIs), focusing on caretakers of children under five in the context of hospital care seeking.

**Methods:**

The study took place in Manhiça, southern Mozambique and used Focused Ethnographic Study tools (FES) including field exercises and interviews.

**Results:**

Understandings of terms used to describe ARIs differed between caretakers and hospital staff. Children's sicknesses that hospital staff diagnosed as ARIs were interpreted by caretakers as intermittent "attacks" of *xifuva*, a permanent, inherent and incurable chest illness. Caretakers thought that it was possible to manage and treat the attacks, which were caused by immediate natural factors such as food or the weather, but not the underlying illness, which was seen as having more indirect and social causes. Explanations of illness could not be neatly separated into pluralistic categories, but were characterised by syncretism, with "lay" and "biomedical" terms and concepts intermingling in practical care-seeking interactions between caretakers and health staff.

**Conclusions:**

Health promotion should take into account the syncretism involved in explanations of ARIs in the context of practical care seeking for children. In doing so, it should draw upon lay interpretations and terminologies in order to stress the importance of seeking hospital care for all *xifuva-*type illnesses as well as seeking care for any subsequent attacks of an already diagnosed *xifuva*. However, this should be undertaken with awareness that the meanings of the terms used in practical care-seeking interactions may change over time. Health communication about ARIs should therefore be ongoing and evidence-based, even if ARIs appear to be well understood.

## Background

In Manhiça, a rural area in Southern Mozambique, malaria is responsible for almost half of all hospital admissions and up to 19% of all deaths among children admitted to hospital [1]. Pneumonia is diagnosed in up to 16% of children admitted to hospital and results in considerable mortality [2]. In this age group, the overlapping signs and symptoms of pneumonia and malaria complicate both diagnosis and treatment. For example, a health worker or parent can easily interpret a fever caused by pneumonia as malaria. If anti-malarials are issued, rather than antibiotics, the child may not recover. Although the Integrated Management of Childhood Illnesses (IMCI) guidelines [3] advocate dual treatment, in practice, health staff tend to be unaware of this and there are no specific education programmes for caretakers.

Whilst it is important for clinical staff to be able to distinguish between the overlapping symptoms of these illnesses in order to correctly diagnose and manage them, caretakers of children also need to be able to recognise the signs and symptoms early so as to seek appropriate medical care [4-7]. Some studies have suggested that caretakers can recognise early respiratory distress (an indicator of an ARI) [4,8,9]. However, other research indicated that caretakers do not clearly recognise the signs and symptoms of ARIs: fast breathing or chest indrawing may not be recognised, or may be wrongly attributed to fever rather than a respiratory infection [10-13].

Poor recognition of signs and symptoms may in part be due to differences between biomedical definitions and classifications of childhood illnesses and local understandings of the overlap of symptoms. Research in South Africa and Uganda, for example, identified many local terms that included the biomedical symptoms of pneumonia and respiratory infections [9,14]. In a recent study in Guinea Bissau, 12% of women and 29% of men had heard of the term 'pneumonia', and only one person out of 620 knew that antibiotics could be used to treat pneumonia [15]. One study in Kenya reported several terms that described chest indrawing, but no local terms to describe rapid breathing [16]. But even if caretakers use biomedical terms to describe ARIs, their meanings may be different to biomedical definitions [13].

Additionally, when caretakers recognise ARI symptoms they may not consider them to be serious, or only see them as serious when a child experiences multiple symptoms [17,18]. For example, in Nigeria, respondents regarded ARI episodes as ordinary coughs and colds caused by cold weather [19]. If illnesses are not perceived as serious, caretakers may delay seeking hospital care [20-23].

Aside from recognition of symptoms and perceptions of severity, other factors affect hospital care seeking. In Ethiopia, mothers recognised the signs of pneumonia, but only 36% sought care from the local health centre [24]. In Bolivia, the rapid onset of an ARI led caretakers to seek assistance from a traditional healer rather than the hospital because the illness was attributed to supernatural factors [11]. Other studies have also shown that the perceived cause of a childhood illness affects where and how treatment is sought [25-28]. Overall, the literature emphasises that to understand caretakers' responses childhood illness it is necessary to explore local perceptions of disease causation.

The research described in this paper explored caretaker interpretations of and responses to childhood ARIs in Manhiça. It complemented a study of the clinical identification of pneumonia and malaria in children under five years old. To the authors' knowledge, no studies of caretaker perceptions of ARIs in Mozambique (and only few in other parts of Southern Africa) have been conducted.

## Methods

### Study setting

This study was conducted in Manhiça District, southern Mozambique, which has an estimated population of 140 000. The population is mainly from the Xironga and Xichangana ethnic groups. Most people are subsistence farmers or work in agricultural cooperatives. Nearly half of all women (47%) and a quarter of all men (24%) are illiterate [29]. Manhiça town has a hospital that serves as the referral health facility for Manhiça District. Adjacent to the hospital is the Manhiça Health Research Centre (Centro de Investigação em Saúde da Manhiça [CISM]), which has been operating a Demographic Surveillance System (DSS) since 1996. Demographic Surveillance Systems have been set up in various sites since the 1960s as a means of tracking longitudinal demographic changes to populations in developing countries. At DSS field sites data on births, deaths (including causes) and migration are collected. Such data are an important resource for evaluating health care interventions and also offer a starting point for new studies [30,31]. The Manhiça DSS produces a permanent population census, updated on a biannual basis, covering an estimated population of around 82 000 inhabitants in an area of around 500 km^2^. Each child living within the DSS study area is issued a unique permanent identification number that describes the geographic localization of his/her household. CISM, in parallel with the DSS, maintains a round-the-clock hospital morbidity surveillance of all outpatient visits and hospital admissions, directly linkable to the DSS. As Manhiça is a DSS and research site it is not representative of Mozambique as a whole. For example, many of the children in the study area have participated in malaria prevention studies, and the related treatment and prevention activities have probably resulted in lower malaria incidence.

The Manhiça District Hospital (MDH) is the referral health centre for a population of around 150 000 people. Utilization of the hospital appears to be high and more than 80% of deliveries in Manhiça District occur at the hospital. Paediatric use of the hospital is high, although people living far away are less likely to attend the hospital. Traditional medicine is a popular resource in Mozambique and is used in response to both health and social problems [32]. In 2004, countrywide, close to 72 000 traditional healers were identified, compared to around 500 medical doctors [33]. However, the extent of traditional medicine use in Manhiça District has not been documented.

### Data collection and analysis

Data were collected in the community and at the hospital (where the clinical study was conducted). Four sites were selected for community data collection: two neighbourhoods close to Manhiça town and the hospital and two sites further from the hospital in more rural neighbourhoods were selected because distance to the hospital might affect hospital usage and understandings of illness. The selection of specific villages was based on the authors' knowledge of the local area and judgement of their representiveness of the population of Manhiça District. To identify potential participants in the community a list of all households in the selected neighbourhoods with children under five years was obtained from the Manhiça DSS database and then randomised using a web-based programme http://www.randomizer.org. Two interviewers then visited these households and asked the caretaker if he or she would be willing to take part in this study.

The caretakers of children admitted to the hospital and participating in the clinical study were recruited as participants at the clinic. The clinical study aimed to better diagnose illnesses with overlapping symptoms (such as pneumonia and malaria) and recruited any child under five displaying clinical symptoms compatible with both diseases (fever or a history of fever and cough, with an increased respiratory rate and concomitant respiratory distress). As, similarly to the clinical research, the social science study, addressed respiratory distress in the context of pneumonia or malaria, the caretakers of children suffering from different illnesses, including malaria and pneumonia, were recruited as participants. Furthermore, the respondents included caretakers whose children died after admission. Caretakers of children enrolled in the clinical study were recruited at the hospital on the days field workers were present.

Several sources were used to record health workers' descriptions of ARIs. Field workers engaged staff involved in the clinical study (nurses and physicians) in informal discussions and carried out observations in the clinic. In addition, the research team was given access to the Case Record Forms (CRFs) used in the clinical study that detailed the clinical diagnoses of children admitted.

Several studies have used Focused Ethnographic Methods (FES) based on the protocol designed for the WHO to investigate local perceptions of childhood ARIs [34,35]. This protocol was designed to rapidly obtain ethnographic information about ARIs for use in local interventions and policy. The FES tools were therefore deemed appropriate for this study as they potentially provide insight into factors affecting care seeking in a quick but in-depth manner. The following data collection tools were utilised: caretaker free listing of common symptoms and illnesses in children under five; hypothetical case scenarios put to caretakers (detailed in Table 1); narrative interviews with caretakers about children's past episodes of ARI/malaria; semi-structured interviews with caretakers of children admitted to hospital with an ARI; and follow-up interviews with caretakers of children admitted to hospital with an ARI.

**Table 1:** Hypothetical case scenarios presented to caretakers

**Scenario A (Severe pneumonia)**
- The child has had a frequent cough for three days
- The child's cough makes it difficult for him or her to breastfeed or eat
- The child's cough makes it difficult for him or her to sleep
- The child's breathing has been faster than usual in the last few hours
- The child's chest makes a noise with every breath
- The child feels warm to the touch
**Scenario B (Mild pneumonia)**
- The child has had an occasional cough for the past three days
- The child is playing and eating normally
- In the last few hours, the child's cough has become more frequent
- The child feels warm to the touch
- The child's breathing seems normal
**Scenario C (Malaria)**
- The child is hot to the touch
- The child is not eating or playing normally
- The child's most recent stools have been more liquid than normal
- The child is paler than usual
- The child is less active than usual
**Scenario D (Malaria plus pneumonia)**
- The child has been warm to the touch for the last three days and is now very hot
- The child has a mild cough
- The child has stopped playing and has become quieter than usual in the last few hours
- The child's breathing has become faster
- The child's chest makes a noise with every breath
- The child's chest is moving very fast
- The child is very pale
- The child has experienced diarrhoea in the last few hours
- The child has vomited in the last few hours

Two research assistants, who were familiar with the area, carried out all interviews in either Changana (the main local language of Manhiça) or Portuguese (the national language). Interviews were recorded, transcribed into Portuguese and subsequently translated into English (if required). KM, who is fluent in Changana, Portuguese and English, carried out translation quality control using a sample of the recorded interviews and transcripts. Data were tabulated using MS Excel and following the techniques described in the FES protocol. Themes from the narrative and follow-up interviews were also identified and extracted using QSR NVivo 2 qualitative data analysis software. The tabulated information and identified themes were then examined and contrasted. Using a grounded theory approach [36], from this initial analysis, a wider hypothesis was generated to interpret the findings. Members of the research team regularly discussed the analysis and emerging hypotheses.

### Ethical considerations

Ethical approval was granted by the National Health Bioethics Committee in Mozambique and by Hospital Clínic's Research Ethics Committee in Barcelona. All participants provided informed consent: caretakers recruited at the hospital signed or thumb printed consent forms; and caretakers recruited in the community signed consent forms or, in the case of illiterate participants, provided verbal consent, which was recorded.

## Results

In total, 57 caretakers of children under five participated in this study: 27 were interviewed at the hospital and 30 were interviewed in the community (see Table 2 for a summary of the study tools used and respondents). Caretakers included children's mothers and other family members (26% [7/27] of caretakers at the hospital) including grandmothers and aunts. Sixty-eight percent of the children whose caretaker was interviewed at the hospital were male. This biased sex ratio reflects that of the clinical study participants (60% male, 40% female) and other studies carried out in Manhiça [1]. Children who participated in the clinical study and whose caretaker was interviewed were, on average, 12 months old (2 to 36 months) and the average age of all the clinical study participants was 14 months (0 to 59 months). An estimated 10% of all children who were enrolled in the clinical study died, and of the 27 whose caretaker was interviewed, one died during the clinical study and one died shortly after (18 days later). The first death was due to sepsis, caused by acute diarrhoea and accompanied by severe respiratory symptoms. According to the verbal autopsy, the second child died from AIDS-related complications. Both children were cared for by their mothers.

**Table 2:** Summary of study tools used

Study Tool	Number of participants
Free listing	18
Hypothetical case scenarios	17 *
Narrative interviews	6
Semi-structured interviews at the hospital	27
Follow-up interviews of hospital participants	19
Informal discussions with clinical study medical doctor	1
Examination of CRFs	30
Observations of health care staff communication with caretakers	NA

*Not all participants were asked about the same number of case scenarios

The results are presented in three sections: firstly, the terms used by caretakers and clinicians for illnesses that children experience are described; secondly, the perceived causes of these illnesses are explored; and, thirdly, the relationship between these concepts, diagnosis, illness identification and care seeking is discussed.

### Describing illnesses and symptoms

The childhood illnesses mentioned most frequently during the free listing exercise are listed in Table 3. The distinction between individual symptoms and illnesses was blurred as some symptoms were described as illnesses and some illnesses were defined solely by a single symptom. For example, *tosse *(literally, cough) can refer to both an illness and a symptom of other illnesses. Two different kinds of diarrhoea (diarrhoea and *rumbsana*) were described as independent illnesses, though clinically they are variations of the same disease. When describing the symptoms of a child with an ARI, caretakers did not discriminate types of breathing difficulty or include nasal symptoms, as medical staff did (either verbally or in the CRFs) (Table 4).

**Table 3:** Illnesses most frequently cited as common in children under five years old

Illness*	Symptoms**
*Malaria *(P)	**Fever, headache, chills**, vomiting, loss of appetite, stomach ache, dizziness, weakness, bile, cough, diarrhoea, painful joints, *constant *fever
*Diarrheia *(P) (Diarrhoea)	**More than 5 stools per day**, vomiting, fever, weakness, defecating a lot
*Rumbsana *(C)	**White stools, constant diarrhoea, bad smell**
*Tosse *(P) (Cough)	**Cough**, snotty nose, fever, sweating, coughing a lot
*Xifuva *(C) (Problems of the chest)	**Breathing badly, noise in chest, cough, deep breathing**, vomiting, fast breathing, fever, loss of appetite
*Mavabyi ya wheti *(C) (Illnesses of the moon)	**Convulsions, falling, being startled/scared**, worms, noises in the belly, not playing, diarrhoea, bloody stools, fever, cough, bad breathing
*Sarampo *(P) *Xitsinana *(C)	**Small spots all over body**, red mouth, red eyes, fever, cough
*Pava-Pava *(C)	**Large Spots**
*Xilala *(C)	**Opening of the head, enlarged head, thin body**, head veins come out, frequent crying

*Illness terms are given in the indicated language: Changana (C) or Portuguese (P) with the literal English translation, if available, underneath.

**Principal symptom(s) in bold type.

**Table 4:** Ways of describing symptoms of ARIs exhibited by children

**Medical staff**
- Lower chest drawing inwards
- Wheezing
- Crepitations (a noise like a plastic bag being crushed or the sound of walking over snow)
- Tubaric murmur (the noise made when one blows into a glass bottle)
- Stridor (a high pitched sound when breathing)
- Nasal flaring
- Runny or blocked nose
**Caretakers**
- Chest problems
- Not breathing well
- Breathing with a lot of effort
- Breathing rapidly
- Breathing loudly
- Breathing more towards the top, but not always

Participants rarely mentioned pneumonia and bronchitis and did not arise in the free listing exercise. *Xifuva *and *tosse *were the terms that most closely resonated with ARIs: *tosse*, an illness itself or a symptom of another illness such as *xifuva*; and *xifuva *(literally, chest) is used as an all-encompassing term to describe any illnesses involving chest or breathing problems. Occasionally, varieties of *xifuva *were also distinguished. The most obviously recognised symptoms were those also seen in asthma (*asma *in Portuguese) to such an extent that sometimes local interviewers and participants used the terms asthma and *xifuva *interchangeably.

Interviewer (I): *You told me that when coming out of the house you thought that your child had asthma, and in the hospital they also said that he had *xifuva, *now I wanted to know if you thought it was really *xifuva *asthma or another type of *xifuva?

Respondent (R): *I thought it was that type of *xifuva, *as the father of this child also has asthma problems; therefore this one has the same *xifuva *problem*

Mother of an infant diagnosed with pneumonia at hospital

In many cases, *xifuva *did share characteristics of medically defined asthma: *xifuva *was seen as chronic, possibly hereditary, characterised by breathing problems and, in some instances, triggered by the weather. However, although symptoms of *xifuva *overlapped with the biomedically-defined asthma, it also included health conditions that are more severe, such as pneumonia and tuberculosis. Little, if any, distinction was made between different illnesses that have breathing problems as a symptom and *xifuva *(or asthma) was often the only term used by community members and some health workers, to describe ARIs, irrespective of 'type'.

### The trajectory and causes of childhood sickness

#### The trajectory of *xifuva *sickness

The data suggest that, in addition to the different meanings of the terms used by biomedical staff and caretakers, perceptions regarding the trajectory of childhood illnesses were also different.

When a sick child attended the hospital, clinical staff interpreted his or her symptoms as resulting from a new sickness event and diagnosed the disease (unless the child had previously been diagnosed clinically as having a chronic disease related to the presenting symptoms). The health workers then endeavoured to treat and cure this disease. However, interviewed caretakers described how the trajectory of several childhood illnesses, including *xifuva*, differed in two ways: firstly, the sickness was present in the child before he or she showed symptoms; and, secondly, a child was not cured after diagnosis and treatment. Other common childhood sicknesses that followed this trajectory were *mavabyi ya wheti *(illness of the moon), *xilala *(head enlarging with depressed fontanel and body thinning) and *xitsinana *(measles).

I: *And, before going to the hospital, what did you think your child had? Did you know that it was bronchitis or you were told that in the hospital?*

R: *Well, I had seen this sickness before. Already, when he was new born he had this problem of coughing a lot...My mother said that this was normal *tosse (*cough*) *that would pass... When I got admitted I did not know, I thought it was *tosse.

Mother of a 10-month-old boy

As part of this trajectory, caretakers saw symptoms as an attack (*ataque*) of an inherent and permanent sickness, which was present whether or not the child had suffered from these symptoms before. In cases where a child became sick with symptoms he or she had suffered previously, the caretaker did not necessarily interpret this as a new sickness event (as a clinician would). Instead, the caretaker attributed the symptoms to the inherent sickness that had caused previous manifestations of the same symptoms. If the symptoms had not occurred previously, this demonstrated the existence of a hidden sickness of which the caretaker was unaware. From this perspective, the hospital was only able to diagnose and treat the attack but not cure the sickness itself, which was seen as incurable. However, once the attack had laid bare the underlying sickness, local care could be sought to manage it and prevent further attacks.

I: And is your child now cured?

*R: I am still fighting in that sense*.

I: How are you fighting, traditionally or in the hospital?

*R: I am fighting traditionally... Asthma is a traditional sickness and it is not cured in the hospital...so I always keep going [to the local healer] for her to receive the treatment, but if I see that my daughter has fever or other complications the only place to take her is the hospital*.

Mother of a child admitted with bronco-pneumonia

#### Causes of children's *xifuva *sickness

The term *xifuva *was also used to describe both aspects of the illness: the underlying sickness or the attack. This explains why the meanings of some symptoms and terms described earlier were blurred. Likewise, when caretakers were explaining the cause of *xifuva*, they had various explanations that referred to either the cause of the attack or to the cause of the sickness.

Explanations of attacks of *xifuva *included changes in temperature or season, chilled food, dust, or smoke. The inherent aspect of *xifuva *was usually attributed to a family history of *xifuva *or certain sexual practices. A child's breathing problems were viewed, for example, as directly related to his or her father's asthma. Sex was described as generating heat that could be transmitted to a child or foetus and have a detrimental effect on his or her health. The closer the bearer of sexual heat was to the child, the worse the effect. So, although any family member could endanger a child's health, mothers carried a particular threat due to her intimate contact with the child during pregnancy and lactation. Sex during lactation carried the further risk of the breast milk becoming contaminated with semen and consequently infecting the child. Childhood illnesses were also caused by improper adherence to prescribed sexual practices and other rituals following the death of relatives (*kutxinga*), abortions or infant deaths. However, as illnesses were not discovered until they had surfaced through an attack, the ultimate cause was often uncertain.

I: How did [the child becoming sick] happen?

*R: When [the father of the child] did *kutxinga *(the following of ritual customs). Also, it might be that he beat the child during that time that he was mourning. I do not know*.

Grandmother of a child previously admitted to hospital with an ARI

#### Causes of other childhood sickness

The ultimate cause of other common childhood sicknesses in Manhiça such as *mavabyi ya wheti *and *xilala*, was attributed to the *nyoka*. The *nyoka *was described as a small snake, which resides, from birth, in a child's stomach. If not correctly managed, the *nyoka *can become active and cause problems. Certain phases of the moon were said to act as a trigger: on crescent nights a child may experience a high fever, diarrhoea and convulsions (*wheti*), which might be diagnosed in the hospital as malaria; and on a full moon, the *nyoka *can pull a child's head, creating a plateau and/or an enlarged head, as well as sometimes shrinking the body (*xilala*), clinically a sign of dehydration and malnutrition. The *nyoka *has been widely reported as a cause of childhood sickness in other parts of Mozambique and southern Africa, particularly for diarrhoea and stomach related problems [37]. However, no evidence was found during this study (or in the literature) that the *nyoka *is invoked in any causal explanation of *xifuva *in children.

The data however suggest that not all childhood sickness was understood as following the same 'inherent' trajectory of *xifuva*. Malaria, for example, was described in biomedically-recognised terms and did not fit this model. Also, a bout of *tosse *(cough) that lasts for a few days was seen as normal and due to environmental factors, such as the weather or dust. For such a bout of *tosse*, biomedical or home-based remedies were used and no further explanation of its cause was required. Only if the *tosse *became prolonged would more intensive treatment be sought and in this case it would be interpreted as part of a *xifuva *attack.

### Identifying and managing attacks of *xifuva*

#### Identifying sickness and determining severity

Caretakers did not readily identify sicknesses or distinguish the severity of symptoms from specific cases. When interviewed at the hospital, caretakers said they could not identify a sickness, gave a different answer to the clinical diagnosis or sometimes they referred to the diagnosis made by hospital staff. In the community, caretakers were very reluctant to identify specific sicknesses or their severity from the case scenarios, despite the research team piloting several different ways of presenting the scenarios. When faced with the scenarios and asked to suggest a possible sickness, 48% (20/41) of caretakers answered, "I don't know". Moreover, perceptions of severity ranged widely within and across the scenarios. Of the participants that proposed a diagnosis when presented with the pneumonia-related case scenarios, 44% (7/16) mentioned *xifuva *or asthma and no one suggested pneumonia.

Caretakers were asked to diagnose the ARI based on a child's symptoms. However, if an ARI was conceptualised as inherent, caretakers identified the symptoms (those presented by the child or described in the scenario) with the attack, not the inherent sickness. To identify the sickness, more information may have been required, such as the child's medical history and family context.

#### The role of the hospital in *xifuva *management

Although they found naming sicknesses difficult, when asked about the case scenarios, caretakers were comfortable discussing care seeking. Irrespective of whether they identified the sickness and its severity, 83% (34/41) of participants said their first response would be to take the child to the hospital. Caretakers interviewed at the hospital reported an average time to care seeking, once symptoms had been identified, of 2.3 days. If the caretaker identified fast breathing, average time to care seeking was shorter (1.7 days). Based on the experiences of 27 children, caretakers interviewed at the hospital recognised 40 symptoms that triggered care: fever (7), cough (6), forced or loud breathing (5), and diarrhoea (4) were the most common. Participants interviewed at the hospital and in the community described how multiple symptoms, rather than specific symptoms, prompted care seeking.

I: Since he was born was he never sick?

*R: No apart from a cough for a few days...I did not do anything because I could control it at home...but I thought about the hospital if the child got a fever*.

Mother of 9-month-old child

Once at the hospital, caretakers were often unsure of the diagnosis or treatment that their children received from hospital staff. Caretakers felt that the health of the child was in the hands of the hospital staff, but they thought the staff were the experts.

I: [Would] you have wanted to change the way your child was treated?

*R: I cannot say anything. I cannot refuse anything [that the hospital offer], as they know how to do their things*.

Mother of a child admitted with convulsions

I: You said that [the hospital staff] gave her saline, what do you think of this treatment?

*R: That I do not know, they are the ones who know*.

Mother of a child admitted with pneumonia

Although caretakers were passive with regard to hospital procedures, they described being well attended and there were few accounts of negative experiences. The hospital was seen as an important source of care for sick children, even though caretakers may not have perceived symptoms as serious and they were unsure of the hospital diagnosis or treatment. To the caretaker, the name that the hospital gives the attack may not be significant as long as the hospital diagnosis of the attack leads to treatment. It also may be more usual to go to hospital when an attack is first seen (and therefore unknown) or has very distressing symptoms. The hospital was a source of care for the management of *xifuva *attacks, but not the inherent sickness.

## Discussion

In Manhiça, caretakers of children and infants interpreted ARI/*xifuva *illnesses as inherent: they are present from birth and manifest themselves in intermittent, visible attacks, remaining latent between attacks, irrespective of treatment. Other childhood illnesses were also understood in this manner, and for these illnesses the *nyoka *was viewed as the underlying cause. Initially, this seemed to be a case of medical pluralism, with local popular "beliefs" about illness existing alongside, and conflicting with, biomedical explanations.

However, interpretations of *xifuva *did not fit easily into either biomedical or popular categories: terms were sometimes used interchangeably, explanations of sickness often included both local and biomedical concepts, and the hospital and non-hospital care was sought in response to attacks. In everyday practice, the distinction between biomedical and local concepts was blurred and health workers often use the vernacular term *xifuva *to discuss ARIs with a caretaker. In such cases it was not always clear whether this was simply a vernacular translation of the biomedical concept to facilitate communication, or whether the health worker shared some of the local meanings of the vernacular term (not unlikely given that both shared the same cultural background). In either case, the use of *xifuva *by the health worker would retain at least some of its vernacular meaning for the caretaker whatever the intention of the health worker.

This ambiguity also made it difficult to use the medical anthropological distinction between illness and disease to analyse responses to ARI/*xifuva*. In the medical social science literature "disease" is what the doctor diagnoses and treats and "illness" is what the patient experiences; the doctor may cure the disease, but for the patient the illness remains unhealed [38]. On one level *xifuva *is the illness and ARI the disease, but this assumes that ARI is unambiguously biomedical and *xifuva *local (and that the two can be separated). However, in Manhiça, in practice, this distinction was not so clear-cut.

Interpretations of *xifuva *in Manhiça also subvert other pluralist distinctions in medical anthropology. So rather than the local causal explanation being either mainly naturalistic (explained in impersonal, systemic terms) or personalistic (caused by a human or supernatural agent) [39], it is both. This in itself is not unusual, as many traditional African aetiologies involve a two-tier explanation, with natural circumstances making the individual susceptible to illness or providing the facilitating circumstances and the conscious agent giving the final push that causes a specific episode at a particular time. In Evans-Pritchard's classic example of Zande explanation, a boy stubs his toe on a tree stump causing a wound, which later becomes infected. The boy explains that the tree stump cased the wound (naturalistic cause), but a witch must have caused the infection, because many similar wounds do not become infected (personalistic cause) [40]. In the case of *xifuva *in Manhiça the model is reversed, with a personalistic factor (sexual practices) providing the background susceptibility and the naturalistic factor (food, dust, weather) providing the trigger for a specific attack.

Given the above, in such situations, it is necessary to move away from pluralism as a model for understanding difference and variation in people's interpretation of sickness. To overcome the bias that disposes us to take logically integrated systems of beliefs as the point of departure [41] a greater focus on the syncretism involved in interpretations of sickness is necessary. So rather than trying to reveal "systems" researchers should focus on studying practice (what people actually do when they are ill or suffer misfortune and how they speak about health and sickness in the practical situations of everyday life, such as care seeking). Health-seeking behaviour is not simply the enactment of "beliefs" within the confines of a "culture" or "system", but a creative process in which we must recognise the role of invention, innovation, and disorder. The anthropologist Johannes Fabian has characterised this approach as "theorising from below" [41].

Syncretism, similar to that observed in Manhiça regarding ARI/*xifuva*, has been reported more widely by medical ethnographers who have moved beyond describing neat systems. For example, in a study of malaria in Tanzania Hausmann-Muela et al.'s informants insisted that witches could not directly cause malaria, but that they could "interfere" with an episode of malaria. They were well aware that malaria parasites, spread by mosquitoes, could be detected in the blood and killed with anti-malarials. But they also thought that witches could make malaria parasites invisible to the hospital microscope. The authors conclude that because of this interaction between two causal models, two types of specialist are needed: first, a traditional healer who removes the power of the witch, and second, a biomedic who eliminate the parasites [42].

All this has implications for the treatment of ARI in Manhiça (and probably other sicknesses in and beyond Manhiça). Health education programmes addressing ARI need to focus on improving communication between health workers and patients/carers by understanding the way in which meanings are shared by ostensibly different sectors of society, and the dynamic way in which variation and interaction occur. Programmes should adopt and integrate vernacular terms and concepts. In Manhiça, this could include strengthening understanding of the different types of *xifuva *attacks (e.g. asthma, pneumonia and bronchitis), using both local and biomedical terminology, so that caretakers are able to distinguish, identify and seek appropriate care for different "types" of *xifuva *attacks, and also stress the importance of hospital care seeking even if *xifuva *symptoms have been seen before. Similarly, explicitly incorporating local concepts and language into hospital care could help to promote the hospital as a source of care for all *xifuva *attacks, not just severe ones.

In Manhiça, the cause of *xifuva *can be uncertain, or attributed to deceased relatives or parental behaviour. It would be interesting to explore more deeply how the diagnosis of *xifuva *relates to blame and responsibility of the causal agent. For example, a study in Bangladesh highlighted how mothers who were not correctly following purdah practices were implicated in the cause of their children's ARIs, and this resulted in them not seeking proper treatment [28]. Social science research has also illustrated how diagnosing childhood illnesses has social implications both within and outside the household [43,44]. This is an area that needs further research, especially in relation to the impact it may have on care seeking.

This concept of health generates questions about the relationship between illnesses in adults and children. This study addressed children's *xifuva *, but in Manhiça *xifuva *also referred to chest problems in adults, such as tuberculosis. Although clinically, adult tuberculosis is a "stand-alone" illness, when patients referred to it as *xifuva *it may be perceived by the patient as fluid and related to other chest problems. This could have implications for the management of illnesses involving respiratory distress in children and adults. The most serious implication of this is that care seeking for a child in respiratory distress may be delayed or mismanaged due to the belief that it is an inevitable attack of an illness that cannot be cured.

The hospital in Manhiça was used as a source of care to manage severe attacks of the illness, but caretakers seemed to be passive with regard to the hospital diagnosis and care. This passivity, seen in both traditional and biomedical healthcare, is perhaps a result of the caretaker's lack of interested in knowing about the diagnosis and care: it is not necessary to understanding medical procedures in order to accept them [45]. In Manhiça, this apparent indifference could simply be a result of a pragmatic approach to illness, which involves making use of all available treatment options (and this would fit with the syncretism described above). It also fits with locally accepted norms relating to the authority of the hospital in dealing with severe attacks of illnesses. In Manhiça, people often spoke of the "law of the hospital", which caretakers and patients invoke to explain their acceptance of hospital procedures even though they do not understand them [46]. The idea of the "law of the hospital" needs further exploration: is it coercive or is it, in fact, a discourse of trust? How is it applied to care seeking for attacks of childhood illnesses? In addition, further research is required into how the inherent aspect of *xifuva *is managed and the implications this may have for promoting hospital care seeking for ARIs.

Many of this study's limitations are associated with the use of the FES study tools. Although some of the study tools were useful, they required extensive adaptation to ensure their appropriateness for use in Manhiça. Adapting the tools reduced the time available for data collection and this had implications for the number of tools that could be used. In some instances, data on topics of interest could not be collected. For example, using the tools did not provide insight into how caretakers talked about or recognised chest indrawing. Moreover, the tools' implicit biomedical categories complicated data collection. For example, the tools were based on the underlying assumption that caretakers distinguished between symptoms and illnesses and were structured such that illness suggestions could only be made on the basis of presenting symptoms. The social and family context of illness and care seeking was not sufficiently incorporated, and elsewhere a similar critique has been made of models in medical anthropology that over-categorise [47].

Due to time and budgetary constraints only caretakers of children were interviewed. Interviewing local healers, professional health care staff and other members of the community would have potentially yielded a deeper understanding of the identification and perceptions of childhood illnesses. The study was also limited by the need for written, rather than verbal, consent, which was mandatory for all research undertaken in Manhiça. This made the participants interviewed at the hospital nervous, in an environment where they were already feeling uncomfortable, and may have affected their responses.

## Conclusion

Caretakers of children in Manhiça perceived ARI as a permanent, inherent illness that is only revealed in occasional discrete attacks. Health promotion addressing childhood ARIs should draw upon this understanding and related terminology, and stress the importance of seeking hospital care for all *xifuva-*type illnesses as well as seeking care for any subsequent attacks of an already diagnosed *xifuva*. However, this should be carried out with awareness that the meanings of the terms used in practical care-seeking interactions may change over time. Health communication about ARIs should therefore be ongoing and evidence-based, even if ARIs appear to be well understood. More research is needed to explore perceptions of the authority of the hospital, the social and political context of diagnosis and childhood sickness and how the inherent aspect of *xifuva *is managed.

## Competing interests

The authors declare that they have no competing interests.

## Authors' contributions

LS participated in the design of the study, initiated the data collection, conducted the analysis, and drafted the manuscript. KM participated in the conception and design of the study, oversaw the data collection, contributed to the analysis, and contributed to the manuscript. QB participated in the design of the study and contributed to the manuscript. SM contributed to the manuscript. CP contributed to the manuscript. AR contributed to the conception and design and of the study, and contributed to the manuscript. RP contributed to the conception and design of the study, contributed to the analysis, and helped draft the manuscript. All authors read and approved the final manuscript.

## Authors' information

Anna Roca was supported by a grant from the Spanish Ministry of Education and Science (Ramón y Cajal: RYC-2008-02777).

## Pre-publication history

The pre-publication history for this paper can be accessed here:

http://www.biomedcentral.com/1471-2458/11/556/prepub
